# Improving Pneumococcal Vaccination Rates in Immunosuppressed Pediatric Patients with Rheumatic Disease

**DOI:** 10.1097/pq9.0000000000000725

**Published:** 2024-05-09

**Authors:** Julia G. Harris, Jordan T. Jones, Leslie Favier, Emily Fox, Michael J. Holland, Amy Ivy, Cara M. Hoffart, Maria Ibarra, Ashley M. Cooper

**Affiliations:** From the * Department of Pediatrics, Children’s Mercy Kansas City, Kansas City, Mo.; † University of Missouri-Kansas City School of Medicine, Kansas City, Mo. 2401 Gillham Road Kansas City, MO 64108

## Abstract

**Background:**

Patients with rheumatic diseases are at a high risk of invasive pneumococcal disease due to immunosuppression. We conducted a quality improvement project, and the first aim was to increase the percentage of patients with systemic lupus erythematosus and mixed connective tissue disease that is up to date on pneumococcal vaccinations from 9.6% to 80% within one year. Subsequently, the second aim was to increase the percentage of patients on immunosuppression with systemic lupus erythematosus, mixed connective tissue disease, juvenile dermatomyositis and systemic vasculitis that is up to date on pneumococcal vaccinations from 62.6% to 80% within one year.

**Methods:**

Two process measures were up-to-date vaccination status on (1) 13-valent pneumococcal conjugated vaccine (PCV13) and (2) 23-valent pneumococcal polysaccharide vaccine (PPSV23). Our outcome measure was being fully up to date on both pneumococcal vaccinations. Interventions included an immunization algorithm, reporting of eligible patients, education, reminders, and pre-visit planning.

**Results:**

There were shifts in the centerline for all quality measures in both phases of this project. The combined pneumococcal vaccination rate for Phase 1 increased from 9.6% to 91.1%, and this centerline was sustained. Pneumococcal vaccination rates also significantly increased for Phase 2: 68.8% to 93.4% for PCV13, 65.2% to 88.5% for PPSV23, and 62.6% to 86.5% for the combined pneumococcal vaccination rate.

**Conclusions:**

Quality improvement methodology significantly increased and sustained pneumococcal vaccination rates in our high-risk, immunosuppressed patients. We continue to prioritize this important initiative to mitigate the risk of invasive pneumococcal disease.

## INTRODUCTION

Patients with childhood rheumatic diseases are at increased risk for serious infections due to immunosuppressive medications. Additional risks may be due to underlying disease-related immune dysfunction. Although immunosuppressed patients cannot receive live virus vaccines, all routine attenuated vaccines, including annual influenza vaccination, are recommended to prevent infections. At the time of this project, the Centers for Disease Control and Prevention (CDC) recommended that immunosuppressed patients receive the 13-valent pneumococcal conjugate vaccine (PCV13) and 23-valent pneumococcal polysaccharide vaccine (PPSV23) to prevent invasive pneumococcal infections.^1–3^ Many patients do not receive PPSV23 from their primary care provider because it is separate from the routine vaccination schedule for healthy children. Additionally, at the time of this project, many immunosuppressed adolescent patients had received the 7-valent pneumococcal conjugate vaccine (PCV7) because PCV13 was not available until 2010.

The European Alliance of Associations for Rheumatology (EULAR) updated their pneumococcal vaccination recommendations after multiple studies concluded that the rates of pneumococcal infections in adult patients with autoimmune inflammatory rheumatic diseases are significantly higher than healthy controls.^4,5^ Additionally, the occurrence and severity of pneumococcal infections in systemic lupus erythematosus (SLE) are worse and lead to higher rates of intensive care unit admission than patients without SLE.^6^ Further, patients with immune-mediated diseases (SLE, Sjögren syndrome, rheumatoid arthritis, polyarteritis nodosa) are at higher risk than those without for developing invasive pneumococcal disease, and the incidence of invasive pneumococcal disease is 13 times higher in patients with SLE.^7,8^

Based on these findings, we sought to vaccinate the highest-risk patients with SLE and mixed connective tissue disease (MCTD), who often have features of SLE and require immunosuppression. Our initial aim was to increase the percentage of patients with SLE and MCTD who are up to date on pneumococcal vaccinations from 9.6% to 80% within 1 year. Then, our second aim was to increase the percentage of patients on immunosuppression with SLE, MCTD, juvenile dermatomyositis (JDM), and systemic vasculitis that is up to date on pneumococcal vaccinations from 62.6% to 80% within 1 year.

## METHODS

This article used the SQUIRE reporting guidelines.^9^

### Context

The project was performed at a free-standing tertiary care pediatric hospital in the Midwest. The rheumatology clinic uses eight pediatric rheumatologists and five nurses and averages more than 4500 ambulatory visits annually. Based on discussions with the hospital’s population health management subsidiary and a network of private pediatric practices, we determined that many general pediatricians were unaware of the recommendations for pneumococcal vaccination in high-risk patients and did not feel accountable for administering these particular vaccines.

### Interventions

**Fig. 1.**
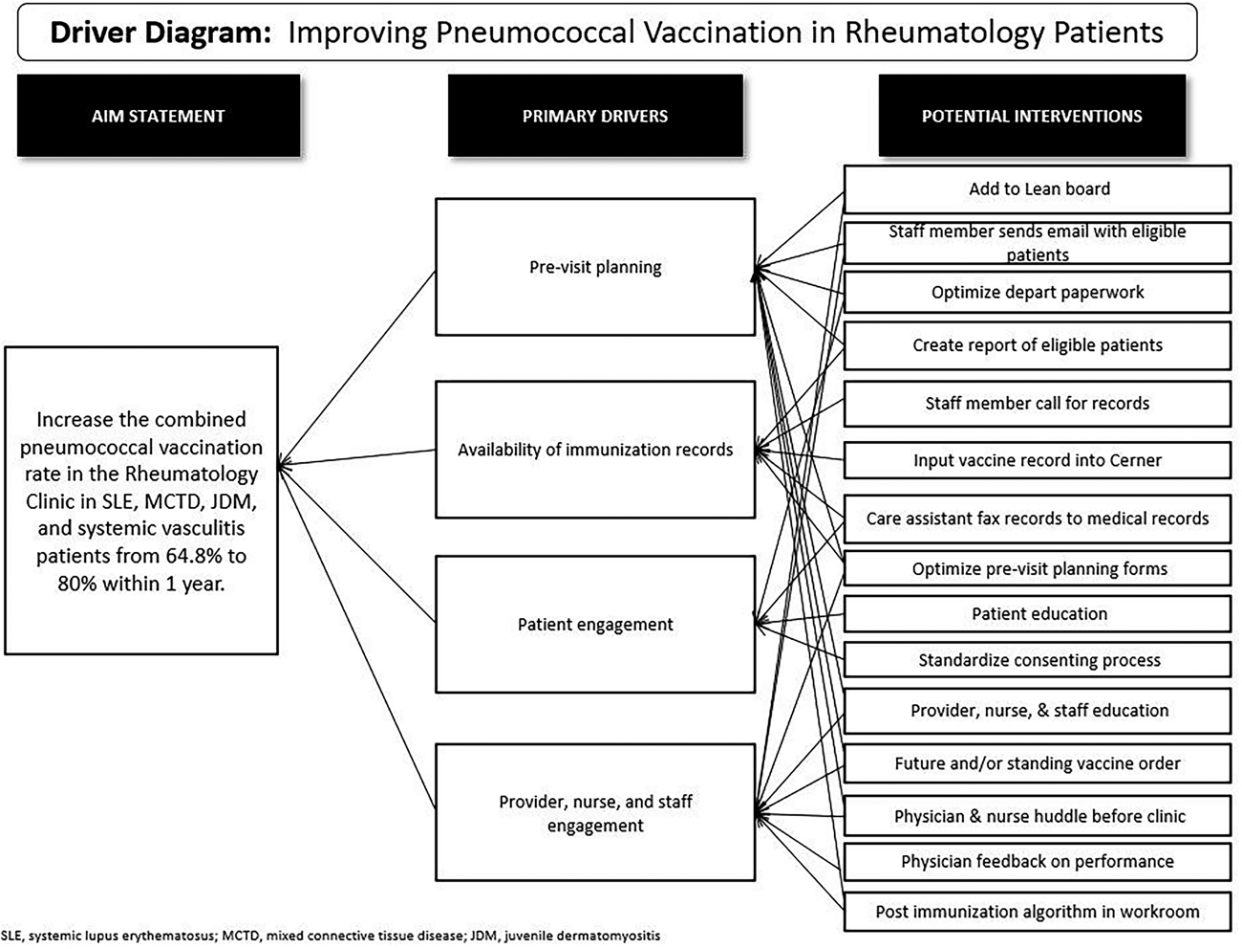
Key driver diagram. Identified key drivers and potential interventions to improve our clinic’s pneumococcal vaccination.

**Fig. 2.**
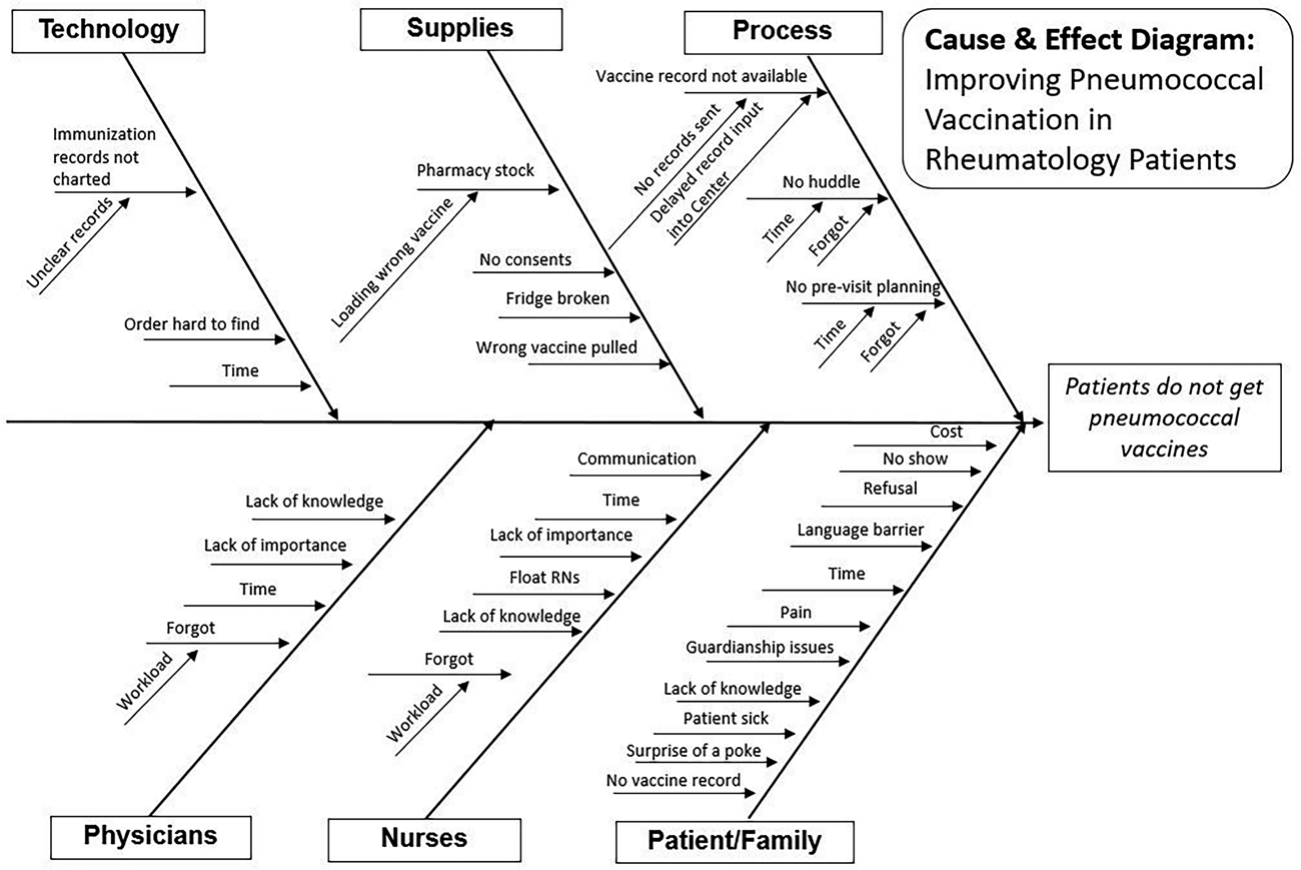
Cause-and-effect diagram. This diagram reviews potential reasons why pneumococcal vaccines are not provided to eligible patients in our clinic.

The rheumatology division established a project team and created a charter as a roadmap. Initial team members included three physicians, a clinic nurse, and a nurse manager. The team made a key driver diagram (Fig. 1) with the primary drivers identified as pre-visit planning, immunization record availability, and engagement of patients, providers, nurses, and staff. A cause-and-effect diagram (Fig. 2) assessed potential barriers to patients receiving a pneumococcal vaccine. The team used quality improvement (QI) methodology to create a process map to identify the steps needed to administer a pneumococcal vaccine in the clinic and to generate a PICK (Possible, Implement, Challenge, Kick Out) chart to prioritize interventions based on ease of change and impact.

Multiple interventions were implemented for this project. The rheumatology division joined a multidivision multidisciplinary team at the hospital, allowing for shared improvement efforts to overcome barriers and reach a broader high-risk population. Physicians and nurses received education about the rationale for pneumococcal vaccines and current vaccine schedule recommendations for immunosuppressed patients. We reviewed a pneumococcal vaccine algorithm and posted this in the common workroom spaces for reference. Pre-visit planning was used to identify high-risk patients eligible for a pneumococcal vaccine, as vaccines were administered during regularly scheduled clinic visits. A report was written based on diagnosis codes, and each week, the administrative assistant emailed physicians potential high-risk patients based on eligible diagnoses. The report was complemented by a broader automated pre-visit planning report created by the hospital’s population health management subsidiary as part of our multispecialty initiative.^10^ The physician retained responsibility for identifying whether vaccination was medically appropriate at the visit based on current medications and prior vaccination status. Further, pneumococcal vaccine project updates and reminders were added to the rheumatology e-newsletter to maintain momentum for the project and provide updates between face-to-face meetings. Throughout the project, performance updates were provided at regular intervals.

### Study of the Intervention

The project team met regularly to discuss the process, vaccination rates, and barriers to vaccination. Multiple plan-do-study-act cycles were done to optimize the process, and immunization rates were assessed temporally related to the interventions. We focused on interventions based on group feedback and assessment to determine the feasibility of implementing the new process within the clinic parameters.

### Measures

The team created two process measures and an outcome measure. One process measure was the percentage of patients with up-to-date PCV13 vaccination status. The numerator varied by age as patients 6 years and older were considered up to date after receipt of one PCV13. In contrast, patients under six may need more than one PCV13, depending on how many PCV7 vaccines they have received previously. Another process measure was the percentage of patients up to date on the PPSV23 based on receipt of one PPSV23 within the past 5 years or two PPSV23 received in their lifetime. The outcome measure was the percentage of eligible patients who were completely up to date on pneumococcal vaccinations, so patients had to satisfy both process measure criteria to fulfill this measure. Initial eligible patients were all SLE and MCTD patients seen for a follow-up visit (phase 1). However, this was later changed to include patients with JDM and systemic vasculitis on immunosuppression (phase 2). We defined an immunocompromised state for our patients largely due to current or planned iatrogenic immunosuppression use and/or functional asplenia in patients with SLE. Exclusions to the measures were established based on the CDC recommendations.^1–3^ Table 1 highlights our measure exclusions.

**Table 1.** Measure Exclusions*

	≤18 years old	≥19 years old
**PCV13 Measure**	Received PPSV23 in the past 8 wk (and have not received PCV13 yet)	Received PPSV23 in the past year (and have not yet received PCV13)
**PPSV23 Measure**	<2 years old	Received PCV13 in the past 8 wk (and have not yet received PPSV23)
	Received PCV13 in the past 8 wk (and have not received PPSV23 yet)	
**Complete Pneumococcal Measure**	<2 years old	Received PCV13 in the past 8 wk (and have not yet received PPSV23)
	Received PCV13 in the past 8 wk (and have not received PPSV23 yet)	Received PPSV23 in the past year (and have not yet received PCV13)
	Received PPSV23 in the past 8 wk (and have not received PCV13 yet)	

* Only one exclusion needed to be excluded from the measure.

PCV13, 13-valent pneumococcal conjugate vaccine.

PPSV23, 23-valent pneumococcal polysaccharide vaccine.

Automated reports allowed for data completeness, and manual chart reviews were performed to validate the patients on the automated report.

### Analysis

Data were gathered via chart review and analyzed biweekly via run charts to assess performance over time. There were separate charts for (1) SLE and MCTD, (2) JDM and vasculitis, and (3) all diagnoses combined for each measure. We assessed for special cause of our outcome measures by the presence of standard control chart rules: (1) shift: 8 or more points in a row above or below the center line and (2) trend: six consecutive points increasing or decreasing.^11^ The final combined outcome measure control chart was annotated to indicate the timing of project interventions.

### Ethical Considerations

Per hospital policy, QI projects do not require review by the institutional review board as they are not considered human subject research.

## RESULTS

Phase 1 included 376 patient encounters with a diagnosis of SLE or MCTD. A range of 2–18 patients were in each biweekly denominator, with an average of 8.5 eligible patients per period. There were 549 eligible patient encounters for Phase 2, 334 with SLE or MCTD and 215 with JDM or vasculitis. The biweekly patient denominator average was 14.8 for Phase 2, ranging from 8 to 31 patients. The age range of our patients was 1–21 years old.

**Fig. 3.**
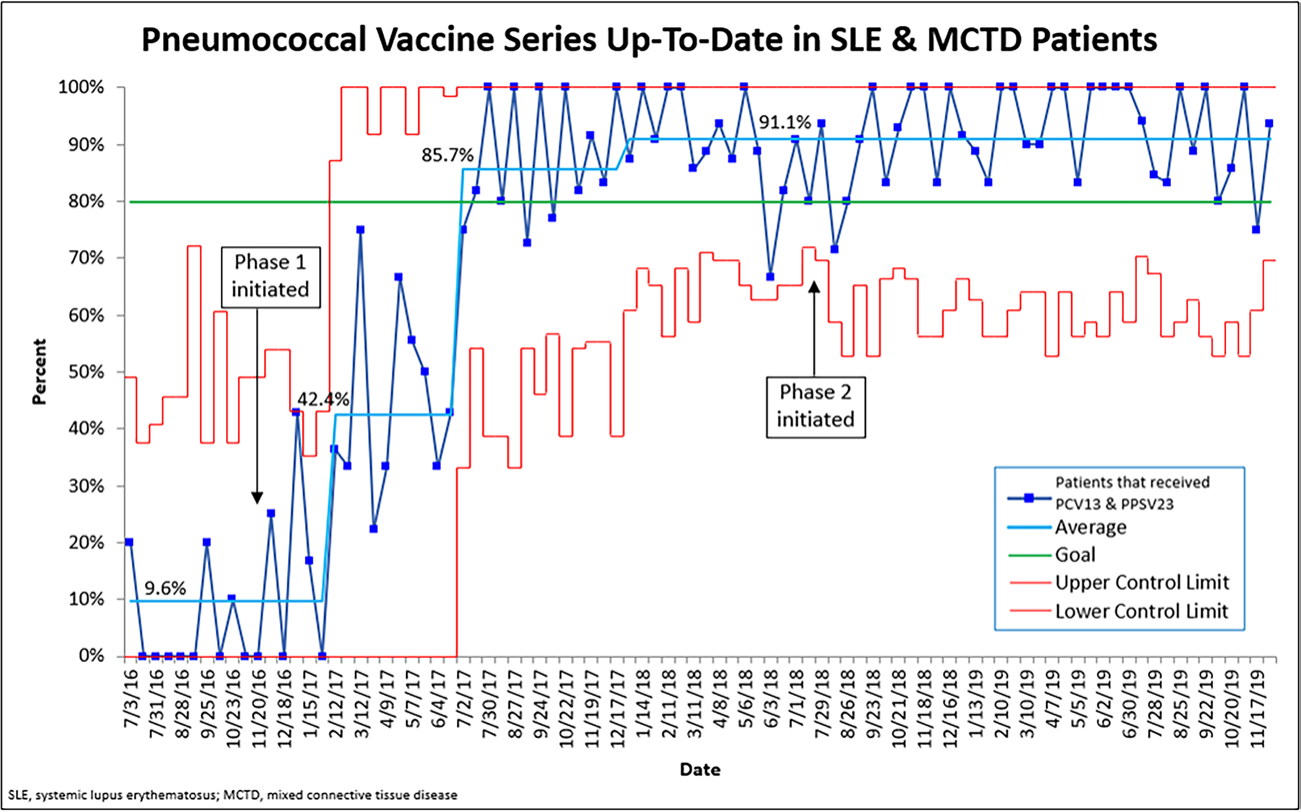
The control chart demonstrates that SLE and MCTD patients are up to date on pneumococcal vaccines. The dark blue line represents the monthly data, the light blue line is the mean, the green line is the goal, and the red lines are the control limits.

For Phase 1, the median vaccination rate of PCV13 for SLE and MCTD patients was 22.5%; after two shifts in the data, the final vaccination rate was 92.3%. The PPSV23 measure had a baseline vaccination rate of 0% and increased to 92.6% after a special cause was present with two shifts. The combined PCV13 and PPSV23 outcome measure for SLE and MCTD patients in Phase 1 is shown in Figure 3. The vaccination rate increased from 9.6% to 91.1% with 3 shifts in the data, and this rate was sustained for two years throughout Phase 2.

**Fig. 4.**
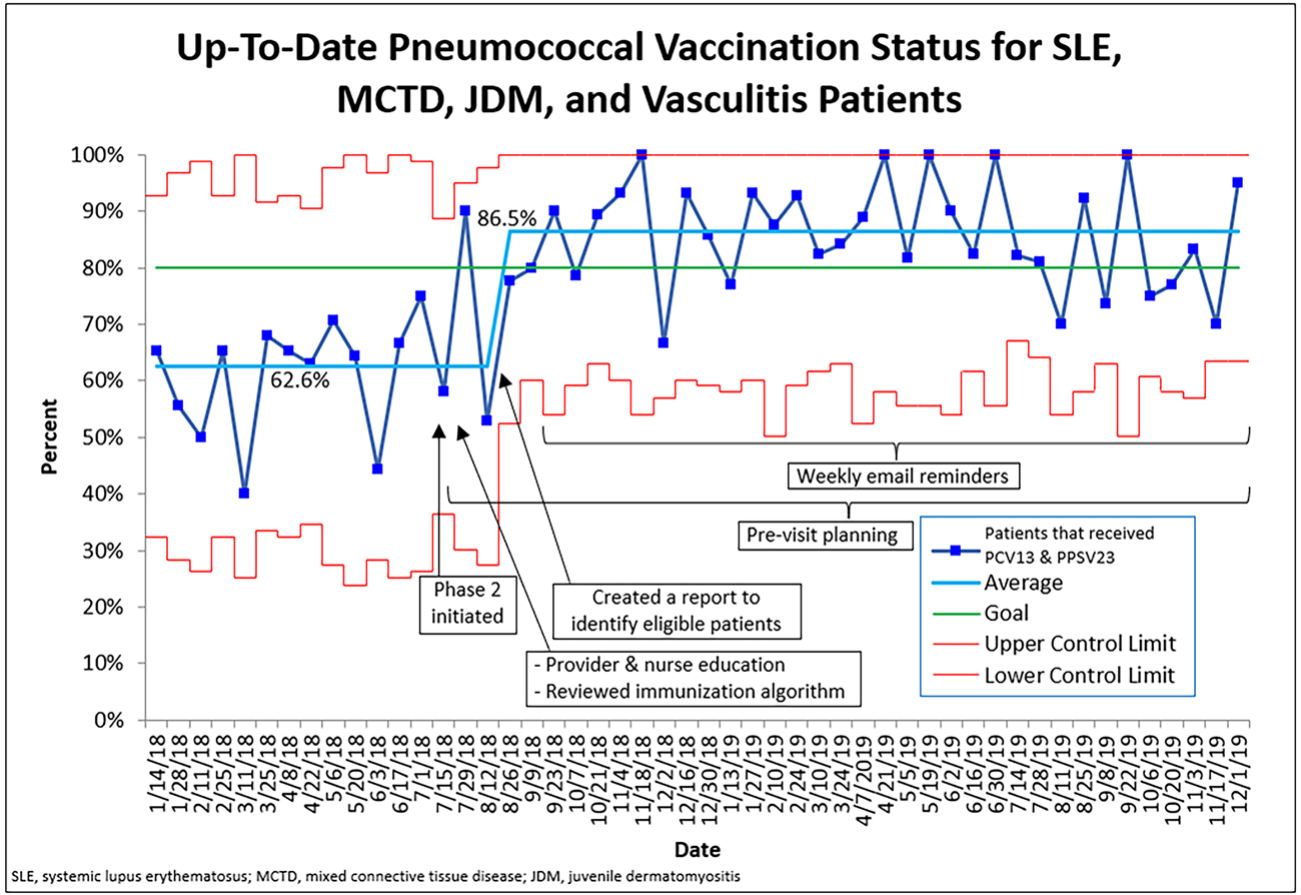
Annotated run chart of SLE, MCTD, JDM, and vasculitis patients that had received both the PCV13 and PPSV23. The dark blue line represents the monthly data, the light blue line is the mean, the green line is the goal, and the red lines are the control limits.

For Phase 2, PCV13 rates for SLE, MCTD, JDM, and vasculitis patients increased from 68.8% to 93.4% with one shift in the data. Special cause was also noted for PPSV23 vaccination rates, which increased from 65.2% to 88.5%. Combined pneumococcal vaccination rates for the high-risk patients demonstrated one shift, going from 62.6% to 86.5%, with the final rate sustained for over one year (Fig. 4). There were no significant known adverse events related to the vaccinations.

## DISCUSSION

### Summary

QI methodology significantly increased and sustained pneumococcal vaccination rates in high-risk, immunosuppressed patients with pediatric rheumatic disease. This multi-year project was successful in both aims and showed markedly increasing pneumococcal vaccination rates for patients at a high risk for invasive pneumococcal disease. The sustainability of these efforts is noteworthy, with vaccination rates holding steady for 2 years for Phase 1 and over 1 year for Phase 2. Further success of this project is attributed to a multidisciplinary team approach with multiple interventions, including pre-visit planning, email reminders, and education. The collaborative nature of the multispecialty initiative at the hospital also allowed for friendly competition, sustained encouragement, and sharing of successes and barriers.

This project could be replicated in other specialty clinics with high-risk populations requiring additional vaccination as the methods are easily accessible. Providers interested in this approach can tailor this project to meet their clinics’ specific needs and patient profiles better. Further, it could be used as an educational tool for providers in the primary care setting to improve vaccination rates in general. In this project, we saw that many primary providers were not aware or comfortable administering additional vaccines to this patient population, and we hope this could be a foundation to build awareness for the vaccines required by high-risk patient populations.

### Interpretation

Many articles have described baseline pneumococcal vaccination rates to highlight the importance of this preventative medicine intervention in high-risk patients. Subesinghe et al assessed infection burden in adult rheumatoid arthritis patients. They found that 7.7% of patients had been hospitalized for a severe infection, and only 44% self-reported receiving a pneumococcal vaccine.^12^ In an internet-based health questionnaire in the United States, 52.5% of immunocompromised adult respondents had received a pneumococcal vaccine.^13^ Before our project, almost a quarter of our patients had received the PCV13, potentially due to routine childhood vaccination; however, few patients were completely up to date on their pneumococcal vaccines. This further highlights the importance of this project.

Other publications have described improvements in efforts to increase vaccination rates in patients with rheumatic diseases with varying levels of success. Garg et al improved pneumococcal vaccination in adult SLE patients from 10% to 59% using pre-visit planning, among other interventions.^14^ Other pediatric rheumatology groups also used pre-visit planning to improve pneumococcal vaccination rates.^15,16^ Other QI strategies to improve vaccination rates have included using an electronic health record alert or prompt, an electronic health record order set, a Lean Six Sigma approach, point-of-care article reminders, and patient letters.^17–23^ Our combined pneumococcal vaccination rate was slightly higher than the 87.3% rate noted by Sivaraman et al. for pediatric patients with SLE.^16^ Their group used a similar approach with an age-based algorithm and pre-visit planning. Our project expanded beyond SLE patients but was less broad than Harris et al, which noted a 33.7% combined pneumococcal vaccination rate for all immunosuppressed pediatric patients with rheumatic disease seen in one tertiary care rheumatology clinic.^15^

### Limitations

Our study has some limitations. Our team intended a complete measure set with a balancing measure assessing the patient’s time in the exam room. However, we realized many contributing factors to the measure, and there was no reliable, automated way to track it. Automated reports were utilized and beneficial, but they relied on staff members to provide information to physicians, which introduced a component of possible human error. However, since the physicians were provided education on the pneumococcal vaccine administration, many did not rely on the automated report to know when to administer a pneumococcal vaccine. A higher reliability intervention would benefit future projects that do not rely on a single person and memory. Our team did not standardly collect race, ethnicity, primary language, or other demographic variables to measure performance. This data would be beneficial to collect to stratify our measures in the future.

Following our project, the CDC issued new pneumococcal vaccine guidance with two additional conjugated pneumococcal vaccines on the market in the United States.^24^ Our project does not reference these new conjugated vaccines, although the QI methodology is still applicable given the new recommendations. Lastly, we did not formally track vaccine refusals, which did occur. It would be helpful in the future to assess why vaccines were not given (ie, vaccine refusals, cost, physician discretion, etc.) as this could lead to further targeted interventions. Although not formally measured, some families received the vaccine at their primary care provider’s office. Cost or insurance barriers were not known factors influencing clinic vaccination decisions. Additionally, physicians did not report disease flare related to the vaccine.

### Conclusions

Using QI methodology, pneumococcal vaccination rates significantly increased and have been sustained in our immunosuppressed pediatric rheumatology patients. We prioritize this important initiative to mitigate the risk of invasive pneumococcal disease in our high-risk patient population.
